# Overexpression of ZNF703 facilitates tumorigenesis and predicts unfavorable prognosis in patients with cholangiocarcinoma

**DOI:** 10.18632/oncotarget.12627

**Published:** 2016-10-13

**Authors:** Keyu Li, Jiabei Wang, Jihua Han, Yaliang Lan, Changming Xie, Shangha Pan, Lianxin Liu

**Affiliations:** ^1^ Department of Hepatic Surgery, The First Affiliated Hospital of Harbin Medical University, Key Laboratory of Hepatosplenic Surgery, Ministry of Education, Harbin, 15001, China

**Keywords:** cholangiocarcinoma, ZNF703, oncogene, tumorigenesis, metastasis

## Abstract

**Background:**

NET (NocA/Nlz, Elbow, Tlp-1) family members have recently emerged as important players in the development of human cancers. Zinc finger protein 703 (ZNF703), locating on chromosome 8 (8p11.23), a member of the NET/Nlz family of zinc finger transcription factors, had been demonstrated to be a much novel oncogene of several malignancies. This study aimed to investigate the expression of ZNF703 in cholangiocarcinoma (CCA) and attempted to elucidate its biological effects in CCA progression.

**Methods:**

The correlation between ZNF703 expression and clinicopathological characteristics of CCA was evaluated through analyzing 85 cases. The biological effects of ZNF703 were investigated both in vitro and in vivo in which proliferation, migration, and invasive potential were mainly explored. Statistical software SPSS 16.0 was used for statistical analyses.

**Results:**

ZNF703 was overexpressed in CCA tissues with subcellular localizations mainly in the nucleus and partly in the cytoplasm or membrane. High expression of ZNF703 was related to tumor location (P=0.002), pathological grading (P=0.024), depth of invasion (P=0.002), distant metastasis (P=0. 011) and AJCC stage (P=0.008). Both in vitro and in vivo studies demonstrated that ZNF703 could potently promote proliferation, migration and invasion throughout the progression of CCA.

**Conclusion:**

ZNF703 can potently facilitate tumor growth and metastasis in many respects throughout the progression of CCA, which may act as an oncogene in CCA and can be considered as a novel potential therapeutic target.

## INTRODUCTION

Cholangiocarcinoma encompass all tumors originating in the epithelium of bile duct, which typically classified as either intrahepatic or extrahepatic cholangiocarcinoma. The incidence of CCA is increasing year by year globally, especially people in Asia and Latin America are more likely to be afflicted with this lethal cancer with a 5-year survival rate less than 5% [1, 2]. Although adjuvant chemotherapy had been confirmed to be relatively effective in improving long-term survival, complete resection is still the only potentially curative method. What challenges most is that the majority of patients are not candidates for surgery due to the presence of advanced disease when confirmed the diagnosis [3, 4]. Hence, it is critical to find new molecular targets underlying CCA proliferation and metastasis in order to develop novel therapeutic strategies [5].

NET family members have recently emerged as important players in the development of multiple structures, from the trachea of fly larvae to the vertebrate eye and some human cancers [6–9]. ZNF703, once known as NocA-like zinc finger protein 1 (Nlz1), a member of the NET family of zinc finger transcription factors, locating on chromosome 8 (8p11.23), had been observed elevations in several types of malignancies including adenocarcinoma of endometrium, gastric cancer, colorectal cancer, breast cancer especially the luminal B subtype [9–13]. Studies had confirmed that ZNF703 as a co-factor of a nuclear complex comprising DDB1 and CUL4 associated factor 7 (DCAF7), prohibitin 2 (PHB2) and nuclear receptor corepressor 2 (NCOR2) could inhibit the transcription of relative genes, regulating cell proliferation, differentiation and cell cycle, finally activate the stem cell-related genes and promote cancer stem cells in luminal B breast cancer [14]. Overexpressed ZNF703 could not only bind with transforming growth factor beta receptor II (TGFBR2) to block the suppressing effect of transforming growth factor beta (TGFβ), but reduce the expression of P15, which served as a cell cycle inhibitor protein, thereby activate the transduction of cell cycle related genes and promote cell proliferation [9, 15]. Besides, overexpressed ZNF703 could even induce the gene expression of lymphoid enhancer-binding factor 1 (LEF1), transcription factor 12 (TCF12), wingless-type MMTV integration site family, member 4 (WNT4) and achaete-scute complex-like 1 (ASCL1) that related to the WNT or NOTCH signaling pathways, to regulate the activity of breast cancer stem cells [16]. Further investigations also confirmed that overexpression of Zeppo1, zinc finger elbow-related proline domain protein 1, mouse ortholog of ZNF703, could modulate proliferation, migration and cell adhesion of tumor cells through repressing the expression of E-cadherin, Wnt and TGF-β reporter, increasing metastases [17].

In an age when molecular targeted therapies for cholangiocarcinoma are so scarce, any potential oncogenes should be deeply explored, in order to improve the development of new targeting agents and the poor prognosis. Data from clinical and experimental researches had revealed that ZNF703 could be considered as a candidate oncogene in some digestive malignancies, while nobody had ever explored its role in the development and progression of cholangiocarcinoma as far as we can investigate. In the present study, we disclosed that ZNF703 was upregulated in CCA and its overexpression could increase the oncogenic potential especially proliferation, migration and metastasis of cholangiocarcinoma.

## RESULTS

### ZNF703 is overexpressed in human cholangiocarcinoma correlating with tumor progression

**Figure 1:**
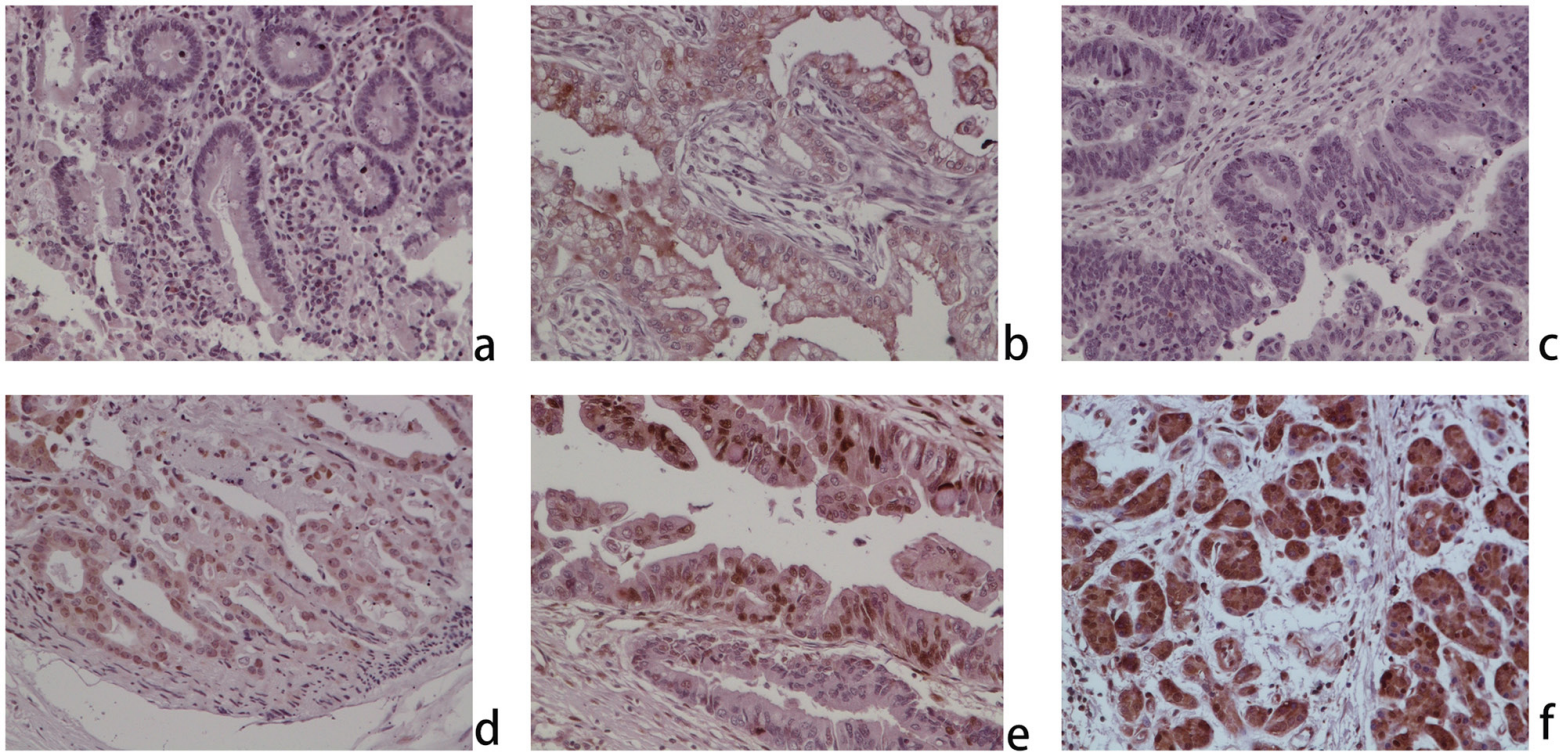
ZNF703 is overexpressed in human cholangiocarcinoma through immunohistochemical analysis (original magnification, x100) **a.** Negative expression in normal cholangitic mucosa; **b.** weak positive nuclear and cytoplasmic expression in normal cholangitic mucosa; **c.** negative expression in CCA; **d.** weak positive nuclear and cytoplasmic expression in CCA; **e.** moderate positive nuclear and cytoplasmic expression in CCA; **f.** positive nuclear and cytoplasmic expression in CCA.

Immunohistochemistry analysis was carried out through samples from CCA microarray and the First Affiliated Hospital of Harbin Medical University, which cover 165 patients including 32 paired para-carcinoma tissues, to investigate the expression of ZNF703 protein and subcellular localization. Results revealed a low expression of ZNF703 in normal para-carcinoma biliary epithelia (8/32, 25.00%), while in contrary, a relatively high expression was observed in 92.12% (152) of all the 165 CCA samples (p<0.001), which mainly localized in the cytoplasm and nucleus. (Figure 1) Moreover, Table 1 summarized the relevance between the expression of ZNF703 and clinicopathological characteristics among 85 CCA patients through comparing age, gender, tumor size, tumor location, pathological grading, invasive depth, lymph node invasion, distant metastasis and American Joint Committee On Cancer (AJCC) stage. Statistical results demonstrated that the expression of ZNF703 was strongly linked to tumor location (P=0.002), pathological grading (P=0.024), depth of invasion (P=0.002), distant metastasis (P=0. 011) and AJCC stage (P=0.008).

**Table 1:** ZNF703 expression and clinicopathological parameters of the 85 CCA patients

	Total	ZES≤4	ZES≥6		
Parameters	n	n (%)	n (%)	χ^2^	P-value
	85	39	46		
Age (years)					
≤60	42	20(47.6)	22(52.4)	0.101	0.751
>60	43	19(44.2)	24(55.8)		
Gender					
Male	45	24(53.3)	21(46.7)	2.138	0.143
Female	40	15(37.5)	25(62.5)		
Tumor location					
Liver	65	32(49.2)	33(50.1)	9.674	0.002
Common bile duct	20	7(35.0)	13(65.0)		
Tumor size (cm)					
>3	59	23(38.9)	36(61.1)	3.698	0.054
≤3	26	16(61.5)	10(38.5)		
Pathological grading					
I-II	44	15(34.1)	29(65.9)	5.108	0.024
III-IV	41	24(58.5)	17(41.5)		
Invasive depth					
T0-T2	60	34(56.7)	26(43.3)	9.555	0.002
T3-T4	25	5(20.0)	20(80.0)		
Lymph node invasion					
Present	27	15(55.6)	12(44.4)	1.491	0.222
Absent	58	24(41.4)	34(58.6)		
Distant metastasis					
Present	7	0(0.00)	7(100.0)	6.467	0.011
Absent	78	39(50.0)	39(50.0)		
AJCC TNM stages					
I-II	60	22(36.7)	38(63.3)	6.978	0.008
III-IV	25	17(68.0)	8(32.0)		

### Expression of ZNF703 is elevated in cholangiocarcinoma cell lines

**Figure 2:**
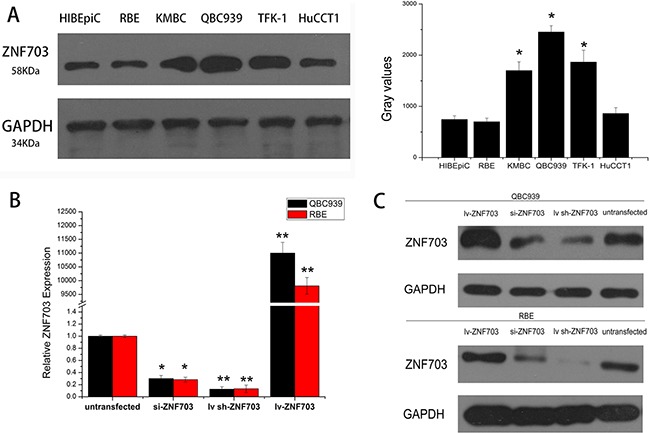
Expression of ZNF703 is elevated in cholangiocarcinoma cell lines **A.** Western blot analysis was selected to detect the different expression level of ZNF703 in the normal HIBEpiC cell lineand other five CCA cell lines, RBE, KMBC, QBC939, TFFK-1 and HuCCT1, which revealed an elevated expression in cholangiocarcinoma cell lines. **B, C.** si-RNA and lentivirus mediated RNA interference were conducted in QBC939 and RBE cell lines, followed by real-time PCR analysis and western blot to confirm the overexpression and inhibition of ZNF703. Data are presented as mean ± SD from six independent experiments, *P <0.05, **P<0.01.

Western blot analysis indicated different levels of ZNF703 protein that expressed not only in normal human intrahepatic biliary cell line but all the five CCA cell lines, in which QBC939 and TFK-1 cell lines as highly aggressive CCA cells expressed more ZNF703 while RBE and HuCCT1 cells with lower capability of invasion and metastasis expressed nearly the same levels of ZNF703, compared to the control normal HIBEpiC cell. (Figure 2A) Based on the results, we proposed a hypothesis that ZNF703 is positive associated with invasion and metastasis in cholangiocarcinoma cells. Hence, QBC939 and RBE cell lines were selected for further investigations to explore whether ZNF703 could change the cell motility in CCA, in which ZNF703 were inhibited or overexpressed through RNA interference technology, for their high or low level of endogenous ZNF703 expression, proliferation and invasion capability, respectively.(Figure 2B and 2C)

### ZNF703 overexpression promotes CCA cells proliferation, migration and invasion

**Figure 3:**
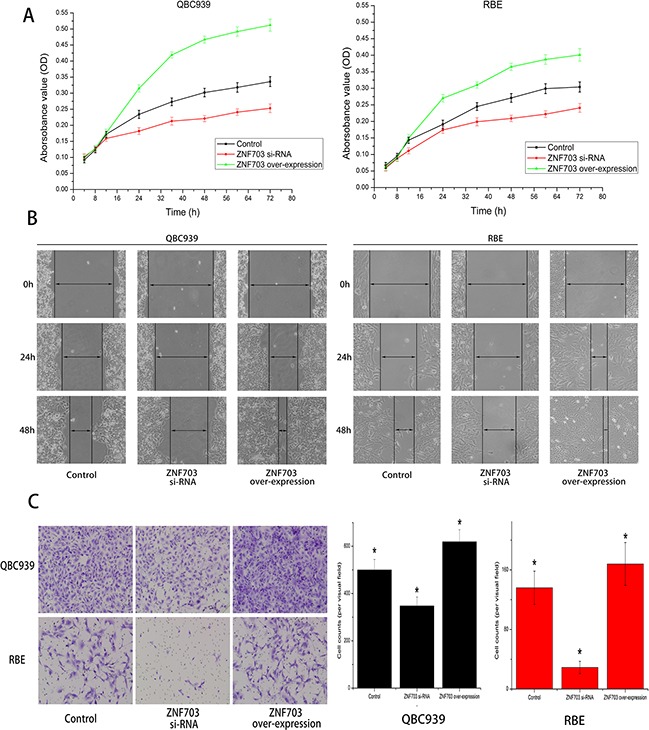
ZNF703 overexpression promotes CCA cells proliferation, migration and invasion in vitro **A.** CCK8 assay were utilized to detect cell proliferation after RNA interference, overexpressing of ZNF703 promoted cell proliferation in both QBC939 and RBE cells, while in contrast, knocking down of ZNF703 inhibited their proliferation abilities, compared to normal CCA cells. Data are presented as mean ± SD from 6 independent experiments, P<0.05 by t-test. **B.** Wound-healing assays of cells treated with blank control, ZNF703 siRNA or ZNF703 overexpression. Migration of cells to cover the scratched wound were visualized at 24 and 48 h with an inverted microscope (original magnification, x100). **C.** Trans-well migration and invasion assays demonstrated that overexpression of ZNF703 significantly increased the migration and invasion capacities compared to the ZNF703 inhibited CCA cells. The number of cells that invaded through the membrane were counted under a Nikon fluorescent microscope with x200 magnification. The results are expressed as the mean ± SD of six independent experiments, *P <0.05.

CCK8 assay were utilized to detect cell proliferation after RNA transduction, as shown in Figure 3A, overexpressing of ZNF703 promoted cell proliferation in both QBC939 and RBE cells, while in contrast, proliferation abilities were reduced after inhibiting ZNF703, compared to normal CCA cells. We also explored the impact of ZNF703 expression on cancer migration through wound-healing method. In line with our expectations, no matter QBC939 or RBE, ZNF703 expression were positively correlated to CCA cells to cover the scratched “wound” in 48 h. (Figure 3B) Furthermore, trans-well migration and invasion assay demonstrated that overexpression of ZNF703 significantly increased the migration and invasion capacities compared to the ZNF703 inhibited CCA cells. (Figure 3C)

### ZNF703 expression promotes CCA tumor growth and metastasis in vivo

**Figure 4:**
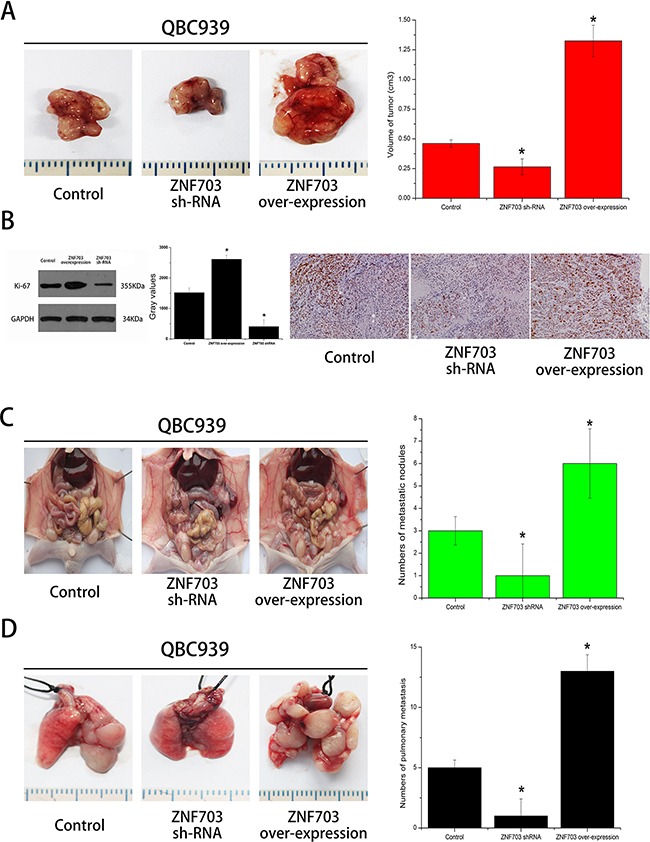
ZNF703 promotes CCA tumor growth and metastasis in vivo **A.** Photomicrographs of xenograft tumors in nude mice. Representative images of a mouse in each group are presented. Tumor volumes in ZNF703 inhibited mice were smaller than those of control mice, while the largest tumors grown in ZNF703 overexpressed mice. **B.** Tumors from different groups were collected for western blot and immunohistochemical analysis in which ki-67 were counted to calculate the proliferation index. Pictures are representative of three independent experiments. **C, D.** The number of tumor masses formed in peritoneal cavity and lungs by QBC939 cells in the ZNF703 inhibited group were much smaller than those formed in control group and overexpressed group, respectively. The results are expressed as the mean ± SD of three independent experiments, *P <0.05.

We further examined the effect of ZNF703 expression on CCA growth through manufacturing a xenograft model in nude mice in which QBC939 cells were utilized. Compared with the control group, ZNF703 overexpressed mice resulted in a significant increase of tumor size while the ZNF703 inhibited mice presented tumors with small size. (Figure 4A) All the tumor tissues were then collected and conducted by immunohistochemistry and western blot analyses, where Ki-67 were selected to assess the proliferative capabilities of each groups. (Figure 4B) These results confirmed a high propensity of ZNF703 expression to promote proliferation of CCA in vivo.

The effects of ZNF703 expression on the metastatic phenotype of CCA were also examined in vivo by implanting QBC939 cells into the peritoneal cavity and tail vein of nude mice, respectively. Necropsy were conducted after 4 weeks and results revealed a significant increase of metastatic nodules in ZNF703 overexpressed mice, while the number of metastatic nodules was reduced in ZNF703 inhibited mice, compared to the control group. (Figure 4C, 4D)

## DISCUSSION

The incidence of cholangiocarcinoma has been increasing steadily over the last few years worldwide, which deserves intense attention especially in Asian countries where the incidence is much higher [19, 20]. Usually genes altered by amplification and concomitant overexpression are considered candidate oncogenes, but amplicons often include several candidates making it difficult to notarize the accurate oncogenes of cholangiocarcinoma [21]. Investigators had sequenced tumors and matching control sample pairs of a large cohort of 103 intrahepatic cholangiocarcinoma patients in China, disclosing 25 significantly mutated genes including eight potential driver genes, gene TP53, KRAS, IDH1, PTEN, ARID1A, EPPK1, ECE2 and FYN [22]. Besides, it had been reported that some other genes like gene pyruvate kinase M2 (PKM2) or stromal cell-derived factor-1 (SDF-1)/CXCR4 et al. could attribute to the progress of CCA [23, 24]. ZNF703 is one of the oncogenic transcription factors that regulates the expression of multiple genes involved in transcription modulation, stem cell regulation, cancer proliferation and invasion [25, 26].

In the present study, we investigated the role of ZNF703 in cholangiocarcinoma, especially into the effect on tumor proliferation, migration and invasion. It's obvious to see from IHC staining that ZNF703 was overexpressed in CCA tissues compared to the matched para-carcinoma ones, with subcellular localizations mainly in the nucleus and partly in the cytoplasm or membrane. Moreover, the high expression of ZNF703 was related to tumor location (P=0.002), pathological grading (P=0.024), depth of invasion (P=0.002), distant metastasis (P=0. 011) and AJCC stage (P=0.008) through analyzing the clinicopathological characteristics of 85 CCA patients. Western blot analysis revealed different amounts of ZNF703 protein that expressed in CCA cell lines in vitro, which maybe positively associated with their different aggressive capabilities. We further demonstrated our hypothesis through conducting RNA interference, ZNF703 was inhibited and overexpressed in QBC939 and RBE cell lines respectively, results indicated a significant reduce of proliferation, invasion and metastasis in QBC cell lines that used to be aggressive CCA phenotype while moderate RBE cell lines displayed to be much aggressive, both in vitro and in vivo. Taken together, ZNF703 could potently facilitate tumor growth and metastasis in many respects throughout the progression of CCA.

Consistent with previous data in which ZNF703 was overexpressed not only in breast but gastric and colorectal cancer and high ZNF703 expression contributed to tumor aggressiveness, our investigations reached much similar conclusions in cholangiocarcinoma. Based on these results, we boldly speculate that ZNF703 could be a novel oncogene of cholangiocarcinoma and even promote tumor progression. However, the mechanisms leading to ZNF703 overexpression are not well established, and it still remains to be explored how the ZNF703 expression or function is regulated [27]. Researchers had identified ZNF703 as a cofactor of a nuclear complex covering DCAF7, PHB2 and NCOR2 through mass spectrometry and pointed out that ZNF703 expression could result in the activation of stem cell-related gene expression leading to an increase in cancer stem cells [14]. Moreover, Reynisdottir and his colleagues had reported that ZNF703 displayed in tamoxifen resistance induced by activation of the Akt/mTOR signaling pathway and downregulation of estrogen receptor alpha, providing a potential mechanism of its action in tumor growth [28].

In general, ZNF703 may act as a transcriptional repressor and affect target genes which mediate various aspects of cancer pathophysiology, such as epithelial-mesenchymal transition, adhesion, motility, proliferation and cancer stem cell renewal, CDH1, cadherin-1, CTTND1, p120-catenin and TGFBR2 [10, 26, 29–31]. Though the potential molecular mechanisms of ZNF703 in carcinogenesis were unclear, we do confirmed its overexpression in CCA tissues and promotion on cancer proliferation, migration and invasion both in vitro and in vivo, which indicate that ZNF703 may result in the discovery of therapeutic targets for better clinical management of cholangiocarcinoma.

## MATERIALS AND METHODS

### Cell lines

The human CCA cell lines KMBC and RBE were obtained from Shanghai Bioleaf Biotech Co., Ltd. (Shanghai, China), QBC939 was a gift from Prof. Shuguang Wang (Third Military Medical University, Chongqing, China), TFK-1 and HuCCT1 were kindly provided by the Cancer Cell Repository from Tohoku University in Japan. The normal human intrahepatic biliary cell line, HIBEpiC, was obtained from ScienCell Research Laboratories (Carlsbad, CA). All the cell lines were cultured in Dulbecco's modified Eagle's medium (DMEM; Gibco BRL, Grand Island, NY, USA) supplemented with 10% fetal bovine serum (FBS, Gibco BRL), penicillin G (100,000 U/L) and streptomycin (100 mg/L; Gibco BRL) at 37°C in a humidified atmosphere containing 5% CO_2_.

### Patients and tumor samples

Formalin-fixed and paraffin embedded tumor samples were purchased from Outdo Biotech Company (HIBD-Ade100PG-01 and HEBD-Ade036PG-01, Shanghai, China) and US Biomax Inc. (B C03119a, Rockville, USA) as tumor microarray, which cover 142 patients with primary human CCA and 9 paired para-carcinoma tissues. Another 23 paired cases were legally collected from the First Affiliated Hospital of Harbin Medical University including 7 paired extrahepatic and 16 paired intrahepatic CCA. Detailed information referring to histological classification, pathological staging, lymphatic or venous invasive conditions were provided in 85 of them including 65 intrahepatic and 20 extrahepatic CCA cases. All the samples were legally sourced for research only with a core diameter and section thickness of 1.5 mm and 4 μm, respectively.

### Immunohistochemistry analysis

Formalin-fixed and paraffin embedded microarrays, patient samples and xenograft samples of nude mice were deparaffinized in Histo-Clear II (SG HS-202, National Diagnostics, USA) and rehydrated through a graded series of ethanol and water followed by heat-induced antigen retrieval in 10 mM sodium citrate buffer (pH 6.0) in a water bath for 15 min at 100°C. After pre-incubating with normal bovine serum, the slides were incubated overnight at 4°C with an optimal dilution (1:100) of a primary polyclonal rabbit antibody against human ZNF703 (GTX107721; GeneTex, Irvine, CA, USA). The slides were sequentially incubated with a biotinylated goat anti-rabbit immunoglobulin G antibody. Reaction products were visualized using diaminobenzidine (DAB; Dako, Carpinteria, CA, USA) with hematoxylin as a counterstain.

ZNF703 expressions were sorted into four categories as negative (0), weak (1) moderate (2) or strong (3) according to the intensity of staining while the percentage of stained cancer cells be counted using the following scale: <5% (0), 5-25% (1), 26-50% (2), 51-75% (3) and >75% (4). The final ZNF703 expression scores (ZES), 0, 1, 2, 3, 4, 6, 9 and 12 were calculated through multiplying these two values. ZNF703 was considered a low expression when ZES were equal or less than 4, while greater than or equal to 6 indicating a high expression [13].

### Western blot analysis

Protein isolation was performed through cell lysis buffer (R0278-50ML, Sigma-Aldrich Co., USA) with their concentrations quantified by Bradford reagent (B6916-500ML, Sigma-Aldrich Co., USA). After boiling, 30 μg of proteins were separated by polyacrylamide gel electrophoresis and transferred to a Hybond ECL nitrocellulose membrane (Amersham Biosciences, Arlington Heights, IL, USA). Membranes were then blocked with 5% low-fat dried milk in phosphate buffered saline containing 0.1% Tween-20 and subsequently incubated for 1 h at room temperature with 1:1,000 dilutions of the primary ZNF703 antibody. Mouse monoclonal anti-GAPDH (KC5G4, KangChen Biotech, Shanghai, China) was used as a loading control. Membranes were then exposed to ECL western blotting substrate (32209, Thermo Fisher Scientific Inc. Waltham, USA) after incubating for 1 h with 1:1,000 dilution of the second antibody in darkness.

### RNA and lentivirus mediated ZNF703 inhibition and overexpression

Logarithmic growth phase cells were plated in 6-well plates at a density of 5×10^5^ cells/ml followed by 24h cultivation. For inhibitive experiments, cells were transfected through a si-ZNF703 (Shanghai GenePharma Company, Shanghai, China) duplexes 5′-CCACACACUUUGGGCCUAAdTdT-3′ (forward) and 5′-dTdTCCACACACUUUGGGCCUAA-3′ (reverse) targeting the 3′UTR of endogenous ZNF703 using Lipofectamine 2000 (Invitrogen) and OPTI-MEM-I in serum-free medium. After culturing for 4h at 37°C containing 5% CO2, the serum-free medium were substituted by normal medium. For overexpression and in vivo studies, cultures were infected with lenti-ZNF703 and lenti sh-ZNF703(Biowot Technologies, Shenzhen, China) containing the entire ZNF703 coding sequence, and sorted by green fluorescent proteins expression to eliminate non-infected cells. The sequences of ZNF703-specific shRNA were 5′–ATGGCAAGAGCCACTTATC-3′. RNA were isolated by RNeasy Mini Kit (74104, QIAGEN, Dusseldorf, Germany) that preserve RNA integrity and expression profiles 24h later. Reverse transcription were then conducted to convert RNA into cDNA, after amplifying the cDNA using ZNF703 specific TaqMan probes (Hs00228155_m1, Thermo Fisher Scientific Inc., Waltham, USA), real-time PCR analysis were utilized to confirm the success of RNA interference.

### Cell Counting Kit-8 (CCK8) assay

Cell proliferation was assessed using the CCK8 assay. Untreated, ZNF703 inhibited and overexpressed CCA cells were seeded in 96-well plates of 1 × 10^4^ cells/ml and incubated overnight in 10% FBS supplemented DMEM for cell attachment. Cells were then incubated for 4h, 8h, 12h, 24h, 36h, 48h, 60h and 72 h before substituting the medium for 200 μl of 10% FBS supplemented DMEM containing 10 μl CCK8 (CK04-01, Dojindo Molecular Technologies, Inc., Japan). Absorbance was then measured at 450nm after 4h of incubation. Six replicates were performed for each experiment.

### Wound healing assay

70 μl of normal, ZNF703 inhibited and overexpressed cell suspension were seeded on wells with Culture-Inserts (Eubio 81176, ibidi, Germany) at a concentration of 5×10^5^ cells/ml. After incubating at 37°C with 5 % CO_2_ for 24 h, the Culture–Inserts were gently removed by sterile tweezers. Complete culture medium was then added, followed by the incubation at 37°C. Wound closure was observed at 0, 6, 12, 24 and 48 h, representative scrape lines were visualized through an inverted microscope and photographed by a Nikon camera. Duplicate wells were examined for each condition with three times replications for each experiment.

### Trans-well migration assay

Invasion was measured using 24-well BioCoat cell culture inserts (BD Biosciences, NJ, USA) with an 8μm porosity polyethylene terephthalate membrane coated with Matrigel Basement Membrane Matrix. Normal, ZNF703 inhibited and overexpressed cells in serum-free medium (2×10^5^ cells/200 μl) were added to the upper chambers while the bottom chambers containing 10% FBS were utilized to supply a chemoattractant. Cells were permitted to migrate through the porous membrane for 24 h at 37°C with 5% CO_2_ while non-migrating cells remained on the upper surface of the filter. Cotton swabs were utilized to gently remove those non-migrating cells before fixing the migrated cells on lower surface with 100% methanol. Migrated cells were then stained by crystal violet and counted under a fluorescent microscope (Nikon Model Eclipse Ni-E, x100). Each experiment was independently performed three times.

### Tumor xenografts in nude mice

All mice were obtained from the laboratory animal center of the Chinese academy of sciences, Shanghai. The experimental protocol was reviewed and approved by the Committee on the Use of Live Animals in Teaching and Research of the Harbin Medical University, Harbin, China. Tumor xenografts were established by standard techniques in 8-week-old nude mice (BALBc nu/nu). Briefly, three mice in similar conditions were chosen as a group, 3 × 10^6^ normal QBC939 cells, lentivirus mediated ZNF703 inhibited and overexpressed QBC939 cells suspended in PBS were injected subcutaneously on the right hip, respectively. Tumor size was measured by a caliper, while tumor volume was calculated as described previously.[18] Mice (n=5 in each group) were sacrificed after feeding under the same conditions for one month and the grown tumor tissues were collected consecutively for protein isolation and IHC analyses.

### In vivo metastatic assay

To evaluate the changes of invasive capabilities induced by ZNF703 expression, BALB/c nude mice, 6-8 weeks of age, were utilized in the studies (n = 8/group). 3 × 10^6^ QBC939 cells, lentivirus mediated ZNF703 inhibited and overexpressed QBC939 cells were injected into the intraperitoneal cavity and tail vein, respectively. Breeding under the same conditions for one month thereafter, all the mice were subtly dissected with representative pictures photographed by a Nikon camera after anesthetization.

### Statistical analysis

Statistical software SPSS 16.0 (Statistical Package for the Social Sciences; SPSS, Inc., Chicago, IL, USA) was used for statistical analyses. All data are expressed as mean values ± standard deviation ($\bar{x}$ ± SD). Analysis of variance and a t-test were used to evaluate statistical significance. The association between ZNF703 expression and CCA clinicopathological characteristics was analyzed using a chi-square (χ^2^) analysis. A value of less than 0.05 (P<0.05) was considered to indicate a statistically significant result.

## References

[1] Siegel RL, Miller KD, Jemal A (2015). Cancer statistics, 2015. CA: a cancer journal for clinicians.

[2] Bergquist A, von Seth E (2015). Epidemiology of cholangiocarcinoma. Best Practice & Research Clinical Gastroenterology.

[3] Koerkamp BG, Wiggers J, Gonen M, Doussot A, Allen P, Besselink M, Blumgart L, Busch O, D'Angelica M, DeMatteo R (2015). Survival after resection of perihilar cholangiocarcinoma—development and external validation of a prognostic nomogram. Annals of Oncology.

[4] Horgan AM, Amir E, Walter T, Knox JJ (2012). Adjuvant therapy in the treatment of biliary tract cancer: a systematic review and meta-analysis. Journal of clinical oncology.

[5] Rizvi S, Borad MJ, Patel T, Gores GJ (2014). Cholangiocarcinoma: molecular pathways and therapeutic opportunities. Seminars in liver disease: NIH Public Access.

[6] Dorfman R, Glazer L, Weihe U, Wernet MF, Shilo B-Z (2002). Elbow and Noc define a family of zinc finger proteins controlling morphogenesis of specific tracheal branches. Development.

[7] Brown JD, Dutta S, Bharti K, Bonner RF, Munson PJ, Dawid IB, Akhtar AL, Onojafe IF, Alur RP, Gross JM (2009). Expression profiling during ocular development identifies 2 Nlz genes with a critical role in optic fissure closure. Proceedings of the National Academy of Sciences.

[8] Ji S-J, Periz G, Sockanathan S (2009). Nolz1 is induced by retinoid signals and controls motoneuron subtype identity through distinct repressor activities. Development.

[9] Holland DG, Burleigh A, Git A, Goldgraben MA, Perez-Mancera PA, Chin SF, Hurtado A, Bruna A, Ali HR, Greenwood W (2011). ZNF703 is a common Luminal B breast cancer oncogene that differentially regulates luminal and basal progenitors in human mammary epithelium. EMBO molecular medicine.

[10] Pereira-Castro I, Costa ÂMS, Oliveira MJ, Barbosa I, Rocha AS, Azevedo L, da Costa LT (2013). Characterization of human NLZ1/ZNF703 identifies conserved domains essential for proper subcellular localization and transcriptional repression. Journal of cellular biochemistry.

[11] Yuan L, Wang P, Lin L, Zhao X, Wang Q (2013). Expression of ZNF703 in Endometrial Adenocarcinoma and its Correlation with Clinicopathological Features of Endometrial Adenocarcinoma. Journal of Practical Obstetrics and Gynecology.

[12] Yang G, Ma F, Zhong M, Fang L, Peng Y, Xin X, Zhong J, Yuan F, Gu H, Zhu W (2014). ZNF703 acts as an oncogene that promotes progression in gastric cancer. Oncology reports.

[13] Ma F, Bi L, Yang G, Zhang M, Liu C, Zhao Y, Wang Y, Wang J, Bai Y, Zhang Y (2014). ZNF703 promotes tumor cell proliferation and invasion and predicts poor prognosis in patients with colorectal cancer. Oncology reports.

[14] Sircoulomb F, Nicolas N, Ferrari A, Finetti P, Bekhouche I, Rousselet E, Lonigro A, Adélaïde J, Baudelet E, Esteyriès S (2011). ZNF703 gene amplification at 8p12 specifies luminal B breast cancer. EMBO molecular medicine.

[15] Orford KW, Scadden DT (2008). Deconstructing stem cell self-renewal: genetic insights into cell-cycle regulation. Nature Reviews Genetics.

[16] Harrison H, Farnie G, Howell SJ, Rock RE, Stylianou S, Brennan KR, Bundred NJ, Clarke RB (2010). Regulation of breast cancer stem cell activity by signaling through the Notch4 receptor. Cancer research.

[17] Slorach EM, Chou J, Werb Z (2011). Zeppo1 is a novel metastasis promoter that represses E-cadherin expression and regulates p120-catenin isoform expression and localization. Genes & development.

[18] Balsari A, Tortoreto M, Besusso D, Petrangolini G, Sfondrini L, Maggi R, Menard S, Pratesi G (2004). Combination of a CpG-oligodeoxynucleotide and a topoisomerase I inhibitor in the therapy of human tumour xenografts. European Journal of Cancer.

[19] Khan SA, Davidson BR, Goldin RD, Heaton N, Karani J, Pereira SP, Rosenberg WM, Tait P, Taylor-Robinson SD, Thillainayagam AV (2012). Guidelines for the diagnosis and treatment of cholangiocarcinoma: an update. Gut.

[20] Rizvi S, Gores GJ (2013). Pathogenesis, diagnosis, and management of cholangiocarcinoma. Gastroenterology.

[21] Santarius T, Shipley J, Brewer D, Stratton MR, Cooper CS (2010). A census of amplified and overexpressed human cancer genes. Nature Reviews Cancer.

[22] Zou S, Li J, Zhou H, Frech C, Jiang X, Chu JS, Zhao X, Li Y, Li Q, Wang H (2014). Mutational landscape of intrahepatic cholangiocarcinoma. Nature communications.

[23] Yu G, Yu W, Jin G, Xu D, Chen Y, Xia T, Yu A, Fang W, Zhang X, Li Z (2015). PKM2 regulates neural invasion of and predicts poor prognosis for human hilar cholangiocarcinoma. Molecular cancer.

[24] Gentilini A, Rombouts K, Galastri S, Caligiuri A, Mingarelli E, Mello T, Marra F, Mantero S, Roncalli M, Invernizzi P (2012). Role of the stromal-derived factor-1 (SDF-1)–CXCR4 axis in the interaction between hepatic stellate cells and cholangiocarcinoma. Journal of hepatology.

[25] Shi Y, Li J, Liu Y, Ding J, Fan Y, Tian Y, Wang L, Lian Y, Wang K, Shu Y (2015). The long noncoding RNA SPRY4-IT1 increases the proliferation of human breast cancer cells by upregulating ZNF703 expression. Molecular cancer.

[26] Zhang X, Mu X, Huang O, Xie Z, Jiang M, Geng M, Shen K (2013). Luminal breast cancer cell lines overexpressing ZNF703 are resistant to tamoxifen through activation of Akt/mTOR signaling. PloS one.

[27] Spellman P, Gray J (2011). A new treasure in the breast cancer gene hunt. Nature medicine.

[28] Reynisdottir I, Arason A, Einarsdottir BO, Gunnarsson H, Staaf J, Vallon-Christersson J, Jonsson G, Ringnér M, Agnarsson BA, Olafsdottir K (2013). High expression of ZNF703 independent of amplification indicates worse prognosis in patients with luminal B breast cancer. Cancer medicine.

[29] Nakamura M, Choe S-K, Runko AP, Gardner PD, Sagerström CG (2008). Nlz1/Znf703 acts as a repressor of transcription. BMC developmental biology.

[30] Bazarov AV, Yaswen P (2011). Who is in the driver's seat in 8p12 amplifications? ZNF703 in luminal B breast tumors. Breast Cancer Res.

[31] Gelsi-Boyer V, Orsetti B, Cervera N, Finetti P, Sircoulomb F, Rougé C, Lasorsa L, Letessier A, Ginestier C, Monville F (2005). Comprehensive profiling of 8p11-12 amplification in breast cancer. Molecular Cancer Research.

